# The power struggle: assessing interacting global change stressors via experimental studies on sharks

**DOI:** 10.1038/s41598-020-76966-7

**Published:** 2020-11-16

**Authors:** Ian A. Bouyoucos, Sue-Ann Watson, Serge Planes, Colin A. Simpfendorfer, Gail D. Schwieterman, Nicholas M. Whitney, Jodie L. Rummer

**Affiliations:** 1 Australian Research Council Centre of Excellence for Coral Reef Studies, James Cook University, Townsville, QLD Australia; 2 PSL Research University, EPHE-UPVD-CNRS, USR 3278 CRIOBE, Université de Perpignan, Perpignan, France; 3 Biodiversity and Geosciences Program, Museum of Tropical Queensland, Queensland Museum, Townsville, QLD Australia; 4 Laboratoire D’Excellence “CORAIL,” EPHE, PSL Research University, UPVD, CNRS, USR 3278 CRIOBE, Papetoai, Moorea French Polynesia; 5 Centre for Sustainable Tropical Fisheries and Aquaculture and College of Science and Engineering, James Cook University, Townsville, QLD Australia; 6 Virginia Institute of Marine Science, William & Mary, Gloucester Point, VA USA; 7 Anderson Cabot Center for Ocean Life, New England Aquarium, Boston, MA USA

**Keywords:** Ecology, Physiology

## Abstract

Ocean warming and acidification act concurrently on marine ectotherms with the potential for detrimental, synergistic effects; yet, effects of these stressors remain understudied in large predatory fishes, including sharks. We tested for behavioural and physiological responses of blacktip reef shark (*Carcharhinus melanopterus*) neonates to climate change relevant changes in temperature (28 and 31 °C) and carbon dioxide partial pressures (*p*CO_2_; 650 and 1050 µatm) using a fully factorial design. Behavioural assays (lateralisation, activity level) were conducted upon 7–13 days of acclimation, and physiological assays (hypoxia tolerance, oxygen uptake rates, acid–base and haematological status) were conducted upon 14–17 days of acclimation. Haematocrit was higher in sharks acclimated to 31 °C than to 28 °C. Significant treatment effects were also detected for blood lactate and minimum oxygen uptake rate; although, these observations were not supported by adequate statistical power. Inter-individual variability was considerable for all measured traits, except for haematocrit. Moving forward, studies on similarly ‘hard-to-study’ species may account for large inter-individual variability by increasing replication, testing larger, yet ecologically relevant, differences in temperature and *p*CO_2_, and reducing measurement error. Robust experimental studies on elasmobranchs are critical to meaningfully assess the threat of global change stressors in these data-deficient species.

## Introduction

Climate change threatens marine ectotherms via myriad global change phenomena^1^. Owing to anthropogenic carbon emissions, the oceans are experiencing changes in physicochemical properties at an unprecedented rate. Unabated climate change projections for the year 2100 suggest that sea surface temperatures will increase by 3–5 °C (ocean warming), and carbon dioxide partial pressures (*p*CO_2_) will increase by ~ 600 µatm (ocean acidification; OA) in pelagic environments; projections are more extreme and variable in coastal environments^2^. These global change phenomena are predicted to affect the fitness and survival of marine ectotherms through reductions in an organism’s physiological oxygen supply capacity^3^. However, alternative hypotheses argue for characterising physiological performance across multiple levels of biological organisation^4^.

Multiple physiological systems are predicted to be affected by global change stressors, including those associated with behaviours. Changes in behaviour in temperate and tropical marine ectotherms are generally associated with simulated OA^5^ conditions in a laboratory setting, such as changes in the strength and direction of lateralisation^6^, predator avoidance^7^, and activity levels^8^. Beyond behavioural performance, well-documented physiological effects of simulated OA conditions include respiratory acidosis (e.g., via reduced extracellular pH) and altered acid–base homeostasis (e.g., via bicarbonate buffering), production of lactate (e.g., via reliance on anaerobic metabolism), and reduced survival^9^. The physiological and behavioural responses to simulated ocean warming conditions have also been well-studied under laboratory conditions; indeed, several thermal tolerance mechanisms are under consideration^3,4^.

Ocean warming and acidification can affect the physiology of marine ectotherms interactively. There is inherent value in quantifying the effects of isolated stressors, but marine environments will be exposed to multiple global change stressors^10^. Interactive effects can be synergistic (i.e., both stressors disproportionately influence effect size), additive (i.e., both stressors contribute individually to effect size), or antagonistic (i.e., exposure to one stressor negates or ‘masks’ the effect of another)^11^. A recent meta-analysis demonstrated that ocean warming and acidification act additively on aerobic scope (i.e., the difference between maximum and standard metabolic rate) in marine ectotherms; yet, mechanisms underlying interactive effects of ocean warming and acidification are unknown^1^. Deleterious, negative interaction effects are, therefore, unpredictable. Despite the complexity of responses observed to date for an impressive diversity of marine taxa, ecological roles, and habitat types, pervasive knowledge gaps remain.

Global change effects on the physiology of large predatory fishes represent a general knowledge gap^12^. As mesopredators and apex predators, these species can exert top-down control in ecosystems^13^. Work with larger specimens is restricted by equipment used to measure physiological performance traits, such as swim flumes and respirometry chambers, and the ability to treat enough replicate individuals; however, studying early life stages can be amenable to available equipment and holding in captivity to ensure that enough replicate individuals are treated. Evidence suggests that early-life stages of teleost fishes (e.g., embryos and larvae) are more sensitive to elevated *p*CO_2_ and temperatures than their adult counterparts^14,15^, thus emphasizing the importance of studying this life-stage to understand a populations’ or species’ vulnerability. Early-life stages of elasmobranch fishes (e.g., sharks) are fully developed at birth/hatch and, therefore, differ considerably from teleost fishes that undergo metamorphosis; yet, the biological ramifications of multiple global change stressors have not been investigated for large predatory elasmobranch fishes at any life stage^16^.

Some shark species rely on shallow, nearshore habitats as nursery areas during early life. These habitats are thought to improve fitness relative to individuals or populations that do not use nursery areas^17^. Neonates can exhibit strong site fidelity to nursery areas^17^, such that these habitats can become ecological traps during extreme conditions such as heatwaves^18^. Indeed, recent studies on volitional activity found that juvenile sharks and rays within nursery areas routinely live at or above temperatures that reduce activity^19^. However, shark species that use such habitats during early ontogeny (e.g., shark/egg nursery areas) have demonstrated resilience to OA-relevant conditions and capacity for reversible acclimation to ocean warming conditions^16^. Conversely, some elasmobranch fishes exhibit unpredictable, deleterious responses to interacting global change stressors^20^. Shark species that derive fitness benefits from shark nursery areas could therefore be at risk if these habitats transition under climate change from nursery areas to ecological traps.

We designed a study with the intention of evaluating responses of reef shark neonates to global change stressors. The first study objective was to identify ex situ physiological and behavioural responses of blacktip reef shark (*Carcharhinus melanopterus*) neonates upon short-term acclimation to various temperature and *p*CO_2_ conditions. To do this, we employed a simple, yet fully factorial, experimental design including two temperature and two *p*CO_2_ levels and three replicate groups of 3–4 sharks (i.e., 9–10 sharks) per treatment. Ecologically relevant temperatures (28 and 31 °C) were selected because they represent average dry and wet season temperatures, respectively^21^. Further, *p*CO_2_ values (650 and 1050 μatm) were selected because they represent a high *p*CO_2_ value that blacktip reef shark neonates currently experience in situ and a mild (+ 400 μatm) acidification scenario^22^, respectively. Brief (i.e., two-week) exposure to static environmental conditions was intended to provide a basic understanding of responses to multiple environmental stressors. Upon exposure, we assessed behavioural (lateralisation and activity) and physiological (hypoxia tolerance, oxygen uptake rates, and acid–base and haematological status) metrics that would encompass the broad range of possible responses observed for elasmobranch fishes and those that had been previously documented in the literature^16^. Because sharks are among the classically ‘hard-to-study’ species in experimental biology, the second and third study objectives were, respectively, to investigate the power of our study design and to quantify the degree of inter-individual variability so that we may make recommendations for future research on similarly difficult species. Robust experimental studies of global change stressors are in need for species like sharks, where slow life-history traits may disproportionately put them at risk of population declines and extirpation in response to global change stressors^16^.

## Results

### Behavioural assays

**Figure 1:**
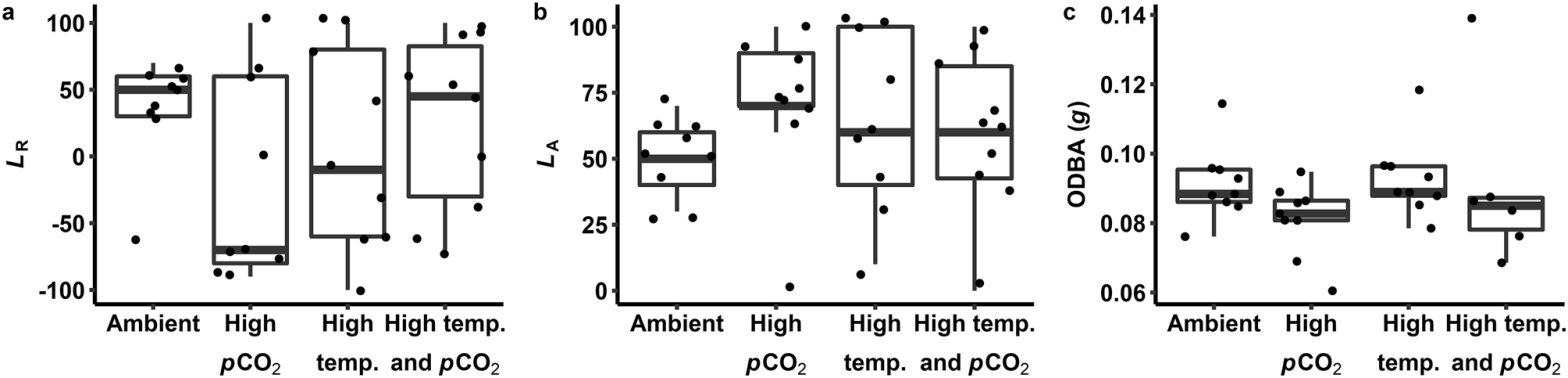
Behaviour of blacktip reef shark (*Carcharhinus melanopterus*) neonates measured under various temperatures and partial pressures of carbon dioxide (*p*CO_2_). Relative (*L*_R_; **a**) and absolute (*L*_A_; **b**) lateralisation indices, and activity levels (overall dynamic body acceleration, ODBA; **c**) were quantified for sharks acclimated to ambient (28 °C and 650 µatm *p*CO_2_), high *p*CO_2_ (28 °C and 1050 µatm *p*CO_2_), high temperature (31 °C and 650 µatm *p*CO_2_), and high temperature and high *p*CO_2_ (31 °C and 1050 µatm *p*CO_2_) conditions for 7–13 days. Dots represent individual observations.

Behavioural metrics were quantified after 7–13 days of exposure to treatment conditions (Table 1). Behavioural lateralisation was quantified after seven days of exposure using a two-way T-maze in untreated water^7^. We were unable to detect treatment effects on the relative lateralisation index (*L*_R_; turning preference scored from − 100 to 100, where positive *L*_R_ indicates a right turning bias; Fig. 1A). Further, the distribution of *L*_R_ (Kolmogorov–Smirnov test, *D* = 0.256–0.556, *p* > 0.100) and variance of *L*_R_ (Bartlett test, *K*^2^ = 3.86, *p* = 0.276) did not differ between treatment groups. We also did not detect treatment effects on the absolute lateralisation index (*L*_A_; strength of lateralisation from 0–100; Fig. 1B) or differences between treatment groups for the variance of *L*_A_ (Bartlett test, *K*^2^ = 5.31, *p* = 0.150).

**Table 1:** Effects of temperature and carbon dioxide partial pressure (*p*CO_2_) on behavioural and physiological metrics in blacktip reef shark (*Carcharhinus melanopterus*) neonates. Linear mixed effects model outputs are presented as the mean and 95% confidence intervals (CI) of effect size of fixed effects terms.

Response	Parameter	Mean	CI
Relative lateralisation index	Intercept	− 17.16	− 61.69 to − 27.78
	High *p*CO_2_	− 17.86	− 66.72 to 32.12
	High temperature	9.05	− 39.15 to 57.59
Absolute lateralisation index	Intercept	56.93	38.93 to 73.73
	High *p*CO_2_	6.85	− 14.08 to 28.24
	High temperature	1.59	− 17.52 to 21.45
Overall dynamic body acceleration	Intercept	0.089	0.081 to 0.099
	High *p*CO_2_	− 0.006	− 0.017 to 0.004
	High temperature	0.004	− 0.007 to 0.015

Volitional activity levels were quantified after 8–13 days of exposure to treatment conditions using accelerometers. To do this, one shark was tagged at a time with an externally attached accelerometer and was then isolated in a 1250 L (1.5 m diameter and 70 cm deep) tank under treatment conditions. After allowing for a two-hour recovery period from the tagging procedure, mean activity levels were calculated over a four-hour window (i.e., 1100–1500) on the day of testing for each shark. We did not detect treatment effects on activity levels, as defined by overall dynamic body acceleration (ODBA, in *g*; Fig. 1C).

### Physiological assays

**Figure 2:**
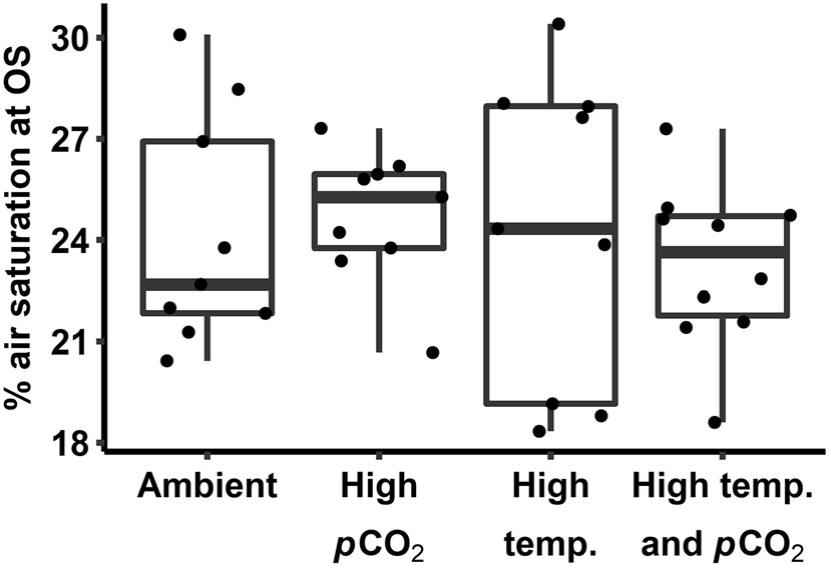
Hypoxia tolerance of blacktip reef shark (*Carcharhinus melanopterus*) neonates measured under various temperatures and partial pressures of carbon dioxide (*p*CO_2_). Hypoxia tolerance was quantified as the percent air saturation at which sharks exhibited the onset of muscle spasms (OS). Sharks were acclimated to ambient (28 °C and 650 µatm *p*CO_2_), high *p*CO_2_ (28 °C and 1050 µatm *p*CO_2_), high temperature (31 °C and 650 µatm *p*CO_2_), and high temperature and *p*CO_2_ (31 °C and 1050 µatm *p*CO_2_) conditions for 14 days. Dots represent individual observations.

Physiological metrics were quantified after 14–17 days of exposure to treatment conditions in the same sharks that underwent behavioural assays (Table 2). Hypoxia tolerance was quantified after 14 days of exposure (Fig. 2). To do this, the oxygen level of an experimental tank containing a single shark was lowered at a constant rate with nitrogen gas. An individual shark’s hypoxia tolerance was recorded as the oxygen level (as a percent of air saturation) at which the shark first exhibited muscle spasms; there were no detectable effects of treatment conditions.

**Table 2:** Effects of temperature and carbon dioxide partial pressure (*p*CO_2_) on physiological metrics in blacktip reef shark (*Carcharhinus melanopterus*) neonates. Linear mixed effects model outputs are presented as the mean and 95% confidence intervals (CI) of effect size of fixed effects terms. Bolded terms represent statistically significant parameters whose confidence intervals do not contain zero.

Response	Parameter	Mean	CI
Hypoxia tolerance	Intercept	24.52	22.57 to 26.58
	High *p*CO_2_	− 0.23	− 2.42 to 2.01
	High temperature	− 0.63	− 2.89 to 1.76
Minimum oxygen uptake rate	Intercept	133.69	109.20 to 158.32
	High *p*CO_2_	22.29	− 6.60 to 52.56
	High temperature	9.29	− 18.23 to 35.22
Maximum oxygen uptake rate	Intercept	360.15	299.76 to 416.29
	High *p*CO_2_	38.75	− 26.53 to 104.84
	High temperature	25.53	− 41.03 to 92.91
Absolute aerobic scope	Intercept	225.26	178.94 to 272.41
	High *p*CO_2_	18.13	− 41.64 to 71.82
	High temperature	17.67	− 32.91 to 69.33
Factorial aerobic scope	Intercept	2.72	2.38 to 3.07
	High *p*CO_2_	− 0.14	− 0.55 to 0.24
	High temperature	− 0.01	− 0.41 to 0.40
Excess post-exercise oxygen consumption	Intercept	369.14	241.92 to 511.92
	High *p*CO_2_	− 84.09	− 248.12 to 78.39
	High temperature	7.86	− 155.66 to 163.72
Recovery time	Intercept	11.75	9.60 to 14.17
	High *p*CO_2_	− 2.28	− 4.99 to 0.32
	High temperature	0.16	− 2.57 to 2.76
Blood pH	Intercept	8.09	8.01 to 8.17
	High *p*CO_2_	− 0.01	− 0.11 to 0.08
	High temperature	− 0.002	− 0.09 to 0.09
Blood lactate concentration	Intercept	3.58	2.65 to 4.53
	**High pCO**_**2**_	− **1.16**	− **2.26 to **− **0.07**
	High temperature	0.12	− 1.05 to 1.24
Haematocrit	Intercept	0.21	0.20 to 0.23
	High *p*CO_2_	− 0.01	− 0.02 to 0.01
	**High temperature**	**0.02**	**0.01 to 0.04**
Haemoglobin concentration	Intercept	0.45	0.30 to 0.60
	High *p*CO_2_	− 0.12	− 0.30 to 0.05
	High temperature	0.05	− 0.11 to 0.23
Mean corpuscular haemoglobin concentration	Intercept	2.07	1.32 to 2.79
	High *p*CO_2_	− 0.49	− 1.32 to 0.31
	High temperature	− 0.01	− 0.90 to 0.74

**Figure 3:**
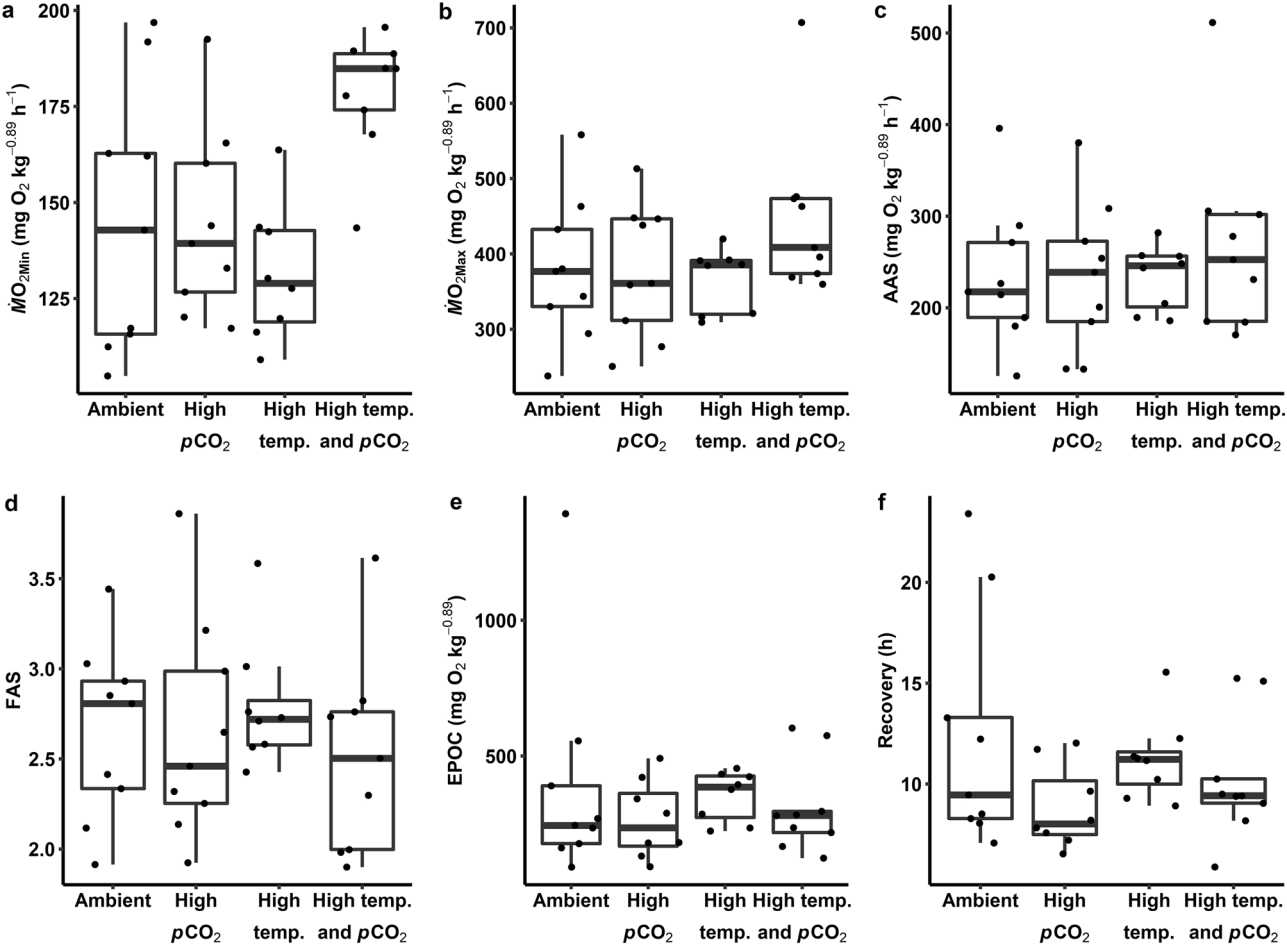
Oxygen uptake rates (*Ṁ*O_2_) of blacktip reef shark (*Carcharhinus melanopterus*) neonates measured under various temperatures and partial pressures of carbon dioxide (*p*CO_2_). Minimum (*Ṁ*O_2Min_; **a**) and maximum (*Ṁ*O_2Max_; **b**) oxygen uptake rates, absolute (AAS; **c**) and factorial aerobic scope (FAS; **d**), excess post-exercise oxygen consumption (EPOC; **e**), and time to recover *Ṁ*O_2_ post-exercise (**f**) were quantified for sharks acclimated to ambient (28 °C and 650 µatm *p*CO_2_), high *p*CO_2_ (28 °C and 1050 µatm *p*CO_2_), high temperature (31 °C and 650 µatm *p*CO_2_), and high temperature and high *p*CO_2_ (31 °C and 1050 µatm *p*CO_2_) conditions for 16 days. Dots represent individual observations.

Oxygen uptake rates (*Ṁ*O_2_, in mg O_2_ kg^−0.89^ h^−1^) were quantified using intermittent-flow respirometry and mass-corrected using an intraspecific scaling exponent of 0.89^23^ (Fig. 3). Our models did not detect significant effects of treatment conditions on minimum oxygen uptake rates (*Ṁ*O_2Min_), maximum oxygen uptake rates (*Ṁ*O_2Max_), absolute aerobic scope (AAS = *Ṁ*O_2Max_-*Ṁ*O_2Min_), factorial aerobic scope (FAS = *Ṁ*O_2Max_·*Ṁ*O_2Min_^−1^), excess post-exercise oxygen consumption (EPOC; the oxygen consumed during recovery, in mg O_2_ kg^-0.89^), or recovery time.

**Figure 4:**
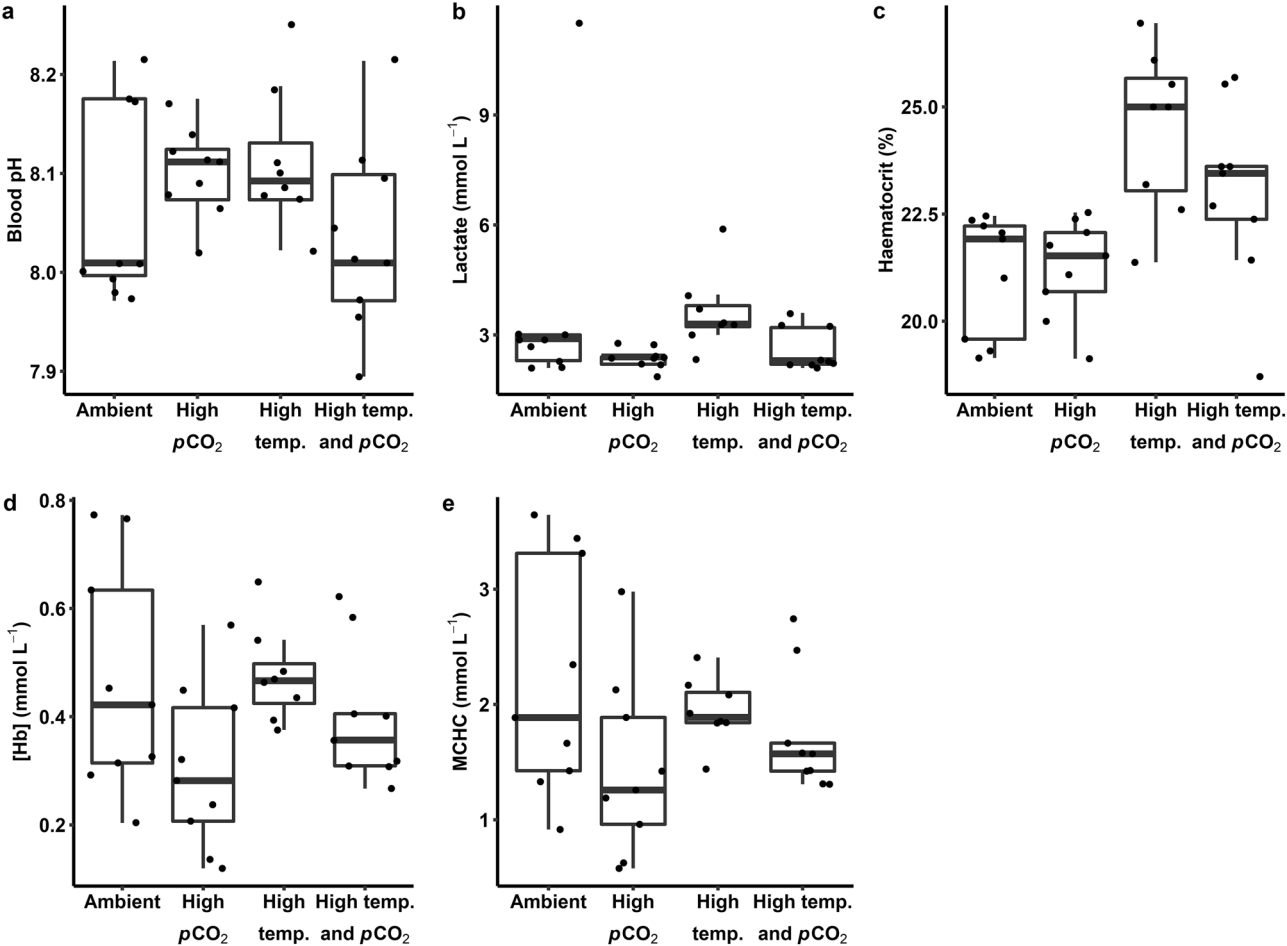
Acid-base and haematological status of blacktip reef shark (*Carcharhinus melanopterus*) neonates measured under various temperatures and partial pressures of carbon dioxide (*p*CO_2_). Blood pH (**a**) and lactate (**b**), haematocrit (Hct; **c**), haemoglobin concentration ([Hb]; **d**), and mean corpuscular haemoglobin concentration (MCHC; **e**) were quantified for sharks acclimated to ambient (28 °C and 650 µatm *p*CO_2_), high *p*CO_2_ (28 °C and 1050 µatm *p*CO_2_), high temperature (31 °C and 650 µatm *p*CO_2_), and high temperature and *p*CO_2_ (31 °C and 1050 µatm *p*CO_2_) conditions for 17 days. Dots represent individual observations.

Acid–base and haematological metrics were quantified from blood samples drawn after sharks had been exposed to treatment conditions for 17 days (Fig. 4). An effect of *p*CO_2_ was detected on whole blood lactate concentration (in mmol L^−1^), where lactate concentration was lower at higher *p*CO_2_ levels. Our models also detected a significant effect of temperature on haematocrit (Hct; the ratio of erythrocyte volume to whole blood volume), where Hct was higher at 31 °C relative to 28 °C. Conversely, no treatment effects were detected for blood pH, haemoglobin concentration ([Hb], in mmol L^−1^), or mean corpuscular haemoglobin concentration (MCHC, in mmol L^−1^).

### Power analysis

A synergistic effect between temperature and *p*CO_2_ was initially detected for *Ṁ*O_2Min_ (observed power = 54%); yet, there was not enough power (i.e., > 80%) to conclude whether there were – or were not – interaction effects. Removing the temperature × *p*CO_2_ interaction term from our models still did not sufficiently increase the power of our models (observed power = 62%). As such, we are unable to claim whether *p*CO_2_ affected lactate. However, there was sufficient power (i.e., 98%) to test for effects of temperature on Hct, and thus we are confident that the observed effect was genuine.

To make recommendations for future study designs, we employed a Monte Carlo simulation based power analysis approach^24^ to estimate the sample size (i.e., replicate groups) required to observe significant treatment effects under our temperature and *p*CO_2_ conditions. We did not consider increasing the number of samples within replicate groups owing to logistical constraints (e.g., number of sharks that could be maintained per tank). First, we identified the number of replicate groups needed to yield enough power to significantly detect our observed treatment effect sizes using *Ṁ*O_2Min,_ which is a representative and well-studied metric across climate change studies in marine ectotherms^1^. At least eight replicate groups (*n* = 96 sharks) per treatment would be needed to detect the effects of elevated *p*CO_2_ (Δ*Ṁ*O_2Min_ = 22.3 mg O_2_ kg^−0.89^ h^−1^) and at least five replicate groups (*n* = 60 sharks) per treatment would be needed to detect the interaction between temperature and *p*CO_2_ (Δ*Ṁ*O_2Min_ = 46.6 mg O_2_ kg^−0.89^ h^−1^). The observed temperature effect was half that of the predicted *p*CO_2_ effect size and small enough such that increases in the number of replicate groups did little to ameliorate power issues.

Finally, using predicted *Ṁ*O_2Min_ values measured for various marine ectotherm taxa (i.e., including teleost and elasmobranch fishes) from a recent meta-analysis^1^, temperature, *p*CO_2_, and interaction effect sizes were manipulated in our *Ṁ*O_2Min_ model to demonstrate an ‘a priori’ experimental design approach. From this, we determined that our original experimental design of including three replicate groups per treatment had sufficient power to detect the predicted interaction effect and the predicted temperature effect. The predicted *p*CO_2_ effect on *Ṁ*O_2Min_ was similarly negligible to the observed temperature effect of the original model.

### Inter-individual variability

Of the all measured physiological traits, lactate concentration, [Hb], MCHC, EPOC, and recovery time exhibited considerable within-group variation (coefficient of variation, CV > 30%). Haematocrit exhibited the lowest within-group variation of any measured trait (CV = 8.4%). After controlling for mass, *Ṁ*O_2Min_, *Ṁ*O_2Max_, and FAS had CVs of 15.3, 17.7, and 18.8%, respectively. Absolute aerobic scope was considerably more variable, with a CV of 27.2%. For behavioural traits, *L*_A_ exhibited tremendous within-group variability (CV = 44.9%). Although ODBA was less variable (CV = 12.3%), this trait exhibited 1.2–1.6-fold variation across treatment groups. Excluding comparisons that were associated through calculations (i.e., MCHC and [Hb], EPOC and recovery, aerobic scope and *Ṁ*O_2Min_ and *Ṁ*O_2Max_), there were no strong correlations (i.e., Pearson’s *r* > 0.80, *p* < 0.001) within or between any measured physiological or behavioural traits.

## Discussion

The purpose of this study was to gain insight into the responses of blacktip reef shark neonates upon exposure to elevated temperatures and *p*CO_2_ levels resembling end-of-century climate change conditions and comment on experimental design and sources of variability. Blacktip reef shark neonates exhibited an increase in Hct upon exposure to 31 °C when compared to sharks maintained at 28 °C. Other statistically significant effects (i.e., lower blood lactate concentrations in sharks maintained at 1050 μatm *p*CO_2_ and a synergistic temperature and *p*CO_2_ effect on *Ṁ*O_2Min_) were not supported by enough power to claim that these were genuine effects of treatment. Our analyses revealed, however, that our study design was sufficient to meaningfully detect the presence or absence of treatment effects, at least on *Ṁ*O_2Min_, for which there are sufficient data to inform such analyses^1^. Instead, due to the variability present in our data, we would need to double the number of replicate groups in our experimental design to be confident that our results (i.e., effects and null effects) were statistically robust. Indeed, this variability likely stems from biological (i.e., inter-individual variability) and experimental sources. We further discuss the potential significance of our findings and highlight logistical considerations for future studies investigating global change stressors in data-deficient and hard-to-study species, such as medium-to-large-bodied sharks.

Temperature acclimation affected the haematological status of blacktip reef sharks. Sharks acclimated to 31 °C for two weeks had a higher Hct than sharks maintained at 28 °C. In teleost fishes, Hct is elevated through a combination of red blood cell swelling, the release of additional erythrocytes into circulation (e.g., from the spleen), and movement of fluid from plasma to interstitial spaces^25^. In blacktip reef shark neonates from the same population examined in this study, Hct increased in response to exercise stress; although, the mechanism underpinning this increase is not yet known^26^. However, Hct did not vary over a 28–31 °C diel temperature range in wild-caught blacktip reef shark neonates from the same population examined in this study^27^. The observed elevation in Hct could compensate for decreases in the affinity of Hb for oxygen that has been documented in vitro with acute warming^21^. Additionally, the increase in Hct at 31 °C may be associated with increases in hypoxia tolerance and thermal tolerance, which has been previously documented for blacktip reef sharks upon four weeks acclimation to 31°C^21^. Previous research in notothenioid fishes and Chinook salmon (*Oncorhynchus tshawytscha*) suggests that Hct is positively associated with critical thermal maximum, a thermal tolerance metric^28,29^. Further, as thermal tolerance and hypoxia tolerance are associated in blacktip reef sharks, an increase in Hct may further support the hypothesis of a common acclimation response and oxygen-dependent mechanism underlying these traits^3^. From an ecological perspective, the consequences of elevated Hct in blacktip reef shark neonates would be experienced in situ during marine heatwaves, such as those that have been occurring with increasing frequency at a similar latitude on the Great Barrier Reef^30^. Elevations in Hct at such high temperatures may lead to exponential increases in blood viscosity that could ultimately affect oxygen transport^31^. Therefore, further investigation into the ecological consequences of haematological responses to warming in reef shark neonates are warranted.

Significant effects of temperature and *p*CO_2_ were detected for several physiological traits. A negative effect of elevated *p*CO_2_ was detected for blood lactate concentration, and a synergistic effect of temperature and *p*CO_2_ was detected for *Ṁ*O_2Min_. However, inter-individual variability in lactate (CV = 45.8%) and *Ṁ*O_2Min_ (CV = 15.3%) was considerable, and the observed power was too low (i.e., < 80%) for us to be confident in these responses. Indeed, acute (i.e., 72-h) exposure to elevated *p*CO_2_ had the opposite effect on blood lactate concentration in blacktip reef shark neonates^22^. At least for *Ṁ*O_2Min_, robust statistical power could have been achieved with additional replicate groups, provided that inter-individual variability did not increase. As variability in mass-corrected *Ṁ*O_2Min_ of blacktip reef shark neonates was within the range reported in teleost fishes^32^, it does not seem likely that additional sampling of similarly sized individuals would increase variability. A few other studies have documented synergistic effects of warming and acidification on *Ṁ*O_2Min_ in teleost fishes; yet, temperature and *p*CO_2_ are predicted to interact antagonistically on *Ṁ*O_2Min_ across a wide array of marine ectotherm taxa^1^. If temperature and *p*CO_2_ truly act synergistically on *Ṁ*O_2Min_ in blacktip reef sharks, this could reflect the increased costs of maintaining acid–base homeostasis in the altered environment. Within an ecological context, environmental oxygen partial pressures (*p*O_2_) in blacktip reef shark neonate habitats around Moorea are routinely supersaturated such that elevations in *Ṁ*O_2Min_ would not be problematic in the context of oxygen availability. Conversely, increased maintenance metabolism costs would necessitate increased foraging success, which is already quite low in the study population^33^ and an increased demand could lead to starvation^34^. Interestingly, supposed changes in *Ṁ*O_2Min_ were not reflected in absolute or factorial aerobic scope, possibly owing to variability in *Ṁ*O_2Min_ and *Ṁ*O_2Max_ and because FAS and AAS estimates were low relative to other fishes^35^. Whilst a lack of correlation between *Ṁ*O_2Min_ and *Ṁ*O_2Max_ across individuals is possible^36^, its absence suggests the possibility of measurement error, likely in *Ṁ*O_2Max_ for which best practice has not been established in sharks^37^. Therefore, additional research is required to confidently detect effects of temperature (alone and interacting with elevated *p*CO_2_^1^) on *Ṁ*O_2Min_ in blacktip reef sharks.

A simple, fully factorial experimental design with two temperature and *p*CO_2_ levels was statistically robust. However, of the 15 metrics and 45 possible treatment effects, only three statistically significant effects were detected with only one supported by adequate statistical power. Minimum *Ṁ*O_2_ is a commonly measured trait in laboratory studies assessing climate change effects in marine ectotherms^1^. In the present study, sufficient power to confidently detect observed temperature, *p*CO_2_, and interaction effects on *Ṁ*O_2Min_ could be achieved with additional replicates; although, a priori power analyses based on a comprehensive meta-analysis across marine ectotherm taxa suggested that our simple experimental design was sufficient^1^. Power analyses for future studies of climate change effects in sharks and rays are limited by very few studies having tested sharks and rays under both temperature and *p*CO_2_ conditions^16^. The issues with statistical power in the present study also inform interpretation of null effects. In other words, does the absence of a significant result imply no effect of treatment, given the observed variability in measured behavioural and physiological traits? A frequentist interpretation (i.e., *p*-value hypothesis testing) of a failure to reject a null hypothesis cannot support this claim; whereas, the approach employed in the present study (i.e., confidence intervals of effect size) can explain whether an effect size overlaps with zero or some practically marginal value^38^. For instance, blacktip reef shark neonates exposed to elevated temperature and *p*CO_2_ conditions were predicted to, on average, exhibit *Ṁ*O_2Min_ values that were 46.6 mg O_2_ kg^−0.89^ h^−1^ higher than sharks exposed to ambient temperature and *p*CO_2_ conditions, but this difference could have been as great as 91.8 mg O_2_ kg^−0.89^ h^−1^ or as little as 0.4 mg O_2_ kg^−0.89^ h^−1^. Practically, this demonstrates that some sharks responded strongly to treatment (i.e., a 62% increase in *Ṁ*O_2Min_); whereas, others did not respond at all. Certainly, the influence of inter-individual variability in traits can be confounding and draw scepticism toward effects and null effects^39^. Identifying and controlling those sources of variability is a critically important endeavour to ensure accurate and reliable estimations of simulated climate change stressor effects in such data deficient groups as sharks and rays.

Blacktip reef shark neonates exhibited inter-individual variation in physiological traits that was not consistent across individuals. Variation in most traits was considerable (i.e., CV > 12%, except for Hct), but within ranges reported for both teleost and elasmobranch fishes. However, interpretation of variability in traits is difficult. Lateralisation exhibits high and context-dependent variability^40^ but has not been widely studied in sharks^41^. Alternatively, for a trait like ODBA for which sources of measurement error in sharks are understood, observed variability is likely due to accelerometers being too large^42^, dorsal fins of neonates not being rigid enough for attachment^43^, and the brevity of monitoring that precluded us from accounting for circadian activity rhythms. Among haematological traits, variability in blood pH, for instance, can result because arterial blood cannot be selectively sampled in elasmobranch fishes using caudal puncture^44^. Variation in *Ṁ*O_2Min_ and *Ṁ*O_2Max_ fell within ranges reported in teleost fishes, whilst variation in aerobic scope was greater^32^. Intrinsic sources of intra-specific variability in metabolic rates (i.e., *Ṁ*O_2Min_, *Ṁ*O_2Max_, AAS) are thought to include genetic, developmental, and biochemical sources of origin, among others^45^. Indeed, genetic variation may account for some variability in these traits; although, blacktip reef sharks around Moorea exhibit inbreeding and low genetic diversity^46^. Extrinsic sources (e.g., local abiotic conditions, food availability) could be at play, given that sub-populations of blacktip reef shark neonates around Moorea occupy such small home ranges^47^ and exhibit variable foraging success^33^. Conversely, the small spatial scale and somewhat homogenous coastline of Moorea^48^ could mean that blacktip reef shark neonate habitats do not differ greatly in abiotic conditions. Further, there were no correlations between physiological and behavioural traits^8^. A lack of consistent associations between traits across individuals meant that sharks could not be coded as ‘high responders’ and ‘low responders’ as a way to control for inter-individual variability^39^. As such, it is difficult to discern whether variation in traits is biologically meaningful, such that climate change ‘winner’ and ‘loser’ phenotypes can be identified.

In conclusion, our findings provide a stepping stone forward but also show the need for more robust and extensive studies to definitively identify the effects of elevated temperature and *p*CO_2_ conditions in a large predatory elasmobranch fish. Indeed, the present study recommends at least a doubling of replicate groups that should be tested in a simple 2 × 2 experimental design, which, in this case, would involve collecting and testing half of the annual neonate population around Moorea, which is not practically feasible. Moving forward, reducing measurement error for traits like activity level and metabolic rate will be paramount in future studies on active shark species and will possibly involve the development and validation of custom equipment, including respirometry systems^49,50^ and data-loggers^42,51^. Controlling for inter-individual variation is likely to be the more challenging endeavour, but this could be addressed by increasing the difference in temperature and *p*CO_2_ conditions between treatments. Statistical approaches that move beyond frequentist null hypothesis testing may also shed light on the interpretation of null results^38^. However, low *p*CO_2_ is difficult to achieve, even at low stocking densities with large fish in field locations^22,52^, and much higher *p*CO_2_ conditions are not relevant to end-of-century climate change projections. Acclimation to higher test temperatures than used in this study can result in mortality^21^, which is an unacceptable endpoint for a protected species like the blacktip reef shark. Indeed, experimentally testing temperature effects on the physiological performance of large-bodied, active sharks has only been accomplished for several species^19,34,51,53^, and testing the effects of elevated *p*CO_2_ has only been accomplished in one study^22^. Therefore, research is critical to provide unequivocal, empirical evidence that yields consensus toward a physiologically-informed framework to inform responsible management for these classically ‘hard-to-study’ species that are already – or will be – threatened by global change.

## Methods

### Ethical approval

All methods were carried out in accordance with relevant guidelines and regulations. Permission to collect, possess, and transport sharks and shark tissues was obtained from the French Polynesian Ministère de la Promotion des Langues, de la Culture, de la Communication, et de l’Environnement (Arrêté N°5129). Ethical approval for all experiments described herein was obtained from the James Cook University (JCU) Animal Ethics Committee (protocol A2394).

### Animal collection

Neonatal blacktip reef sharks (n = 37, total length = 569.2 ± 31.9 mm, mass = 1.0 ± 0.2 kg; data presented are means ± standard deviation unless noted otherwise) were collected from putative shark nursery areas around the island of Moorea, French Polynesia from October 2018 through January 2019. Sharks were fished at dusk using monofilament gill-nets (50 m by 1.5 m, 5 cm mesh size), and were transported in 200 L coolers of aerated seawater to a laboratory facility. We marked sharks for identification with uniquely coloured spaghetti tags (Hallprint, Hindmarsh Valley, SA, Australia) and passive integrated transponders (Biolog-id SAS, Paris, France). Animals were held under natural photoperiod in flow-through, 1250 L circular tanks (3–4 sharks per tank) and were fed ad libitum every second day with fresh tuna (*Thunnus* spp.) except for 24–48 h of fasting prior to testing. Feeding was monitored to ensure that all sharks ate during each feeding event. On average, sharks gained mass and did not change in body condition while in captivity. Following experimentation, after 21–34 days in captivity, sharks were released in good condition at their original capture site.

### Experimental design

Sharks were acclimated to combinations of temperature (28 and 31 °C) and *p*CO_2_ (650 and 1050 µatm) that are representative of ambient conditions of Moorea’s lagoon and projected end-of-century *p*CO_2_ in a fully factorial design (Table 3). Acclimation to temperatures above 31 °C is associated with mortality, and thus temperatures higher than 31 °C were avoided^21^. Three replicate groups of 3–4 sharks were tested at each temperature and *p*CO_2_ combination, and up to four replicate tanks could be tested at any given time. Experiments were conducted between November 2018 and January 2019, and replicates within treatments were conducted across the entire study period. Behavioural assays were conducted at seven days (lateralisation) and 8–13 days (activity levels) of acclimation, as behavioural responses are apparent after several days at high *p*CO_2_^5,16^. Physiological assays were conducted after 14 days (hypoxia tolerance), 16 days (oxygen uptake rates), and 17 days (acid–base and haematological status) of acclimation.

**Table 3:** Experimental treatment seawater chemistry. Values are presented as means ± standard deviation. Temperature, pH on the National Bureau of Standards scale (pH_NBS_), salinity, and total alkalinity were measured directly and used to calculate carbon dioxide partial pressures (*p*CO_2_) in CO2SYS^54^.

Target *p*CO_2_	Target Temperature	Temperature (°C)	pH_NBS_	Salinity	Total Alkalinity (µmol kg SW^−1^)	*p*CO_2_ (µatm)
650 µatm	28 °C	28.1 ± 0.2	8.01 ± 0.02	37 ± 1	2354 ± 14	657 ± 50
650 µatm	31 °C	30.7 ± 0.3	8.01 ± 0.01	38 ± 1	2351 ± 9	637 ± 5
1050 µatm	28 °C	28.0 ± 0.1	7.86 ± 0.01	38 ± 1	2337 ± 7	1015 ± 11
1050 µatm	31 °C	30.8 ± 0.2	7.81 ± 0.07	37 ± 0	2358 ± 32	1150 ± 76

### Seawater chemistry

After habituation, temperature conditions were achieved in 0.5 C d^−1^ increments using aquarium heaters (Jager 300w, EHEIM GmbH & Co KG, Deizisau, Germany) or chillers (TK-1000/2000, TECO S.r.l., Ravenna, Italy)^21^. Elevated *p*CO_2_ conditions were achieved once target temperatures were reached. Unique header tanks (288 L) for each *p*CO_2_ treatment tank were dosed with CO_2_ using a pH controller system (AT Control System, AB Aqua Medic GmbH, Bissendorf, Germany) set to pH values on the National Bureau of Standards scale (pH_NBS_).

Four physicochemical parameters were measured to calculate seawater *p*CO_2_: pH_NBS_, total alkalinity, temperature, and salinity^55^. Holding tank pH_NBS_ was measured daily with a handheld meter (Seven2Go Pro, Mettler-Toledo GmbH, Greifensee, Switzerland) and was calibrated as needed with pH_NBS_ 4 and 7 buffer solutions. Data-loggers (DS1922L, Maxim Integrated Products, Inc., San Jose, CA, USA) recorded temperatures hourly. Salinity was measured daily with a handheld refractometer. Total alkalinity (A_T_, µmol kg seawater^−1^) of holding tank water was measured via open-cell Gran titration following standard operating procedure 3b^56^. Seawater samples (50 mL) were dosed with 0.1 M HCl in 0.1 mL increments, and A_T_ was calculated using custom R script (F. Gazeau, unpublished data). The titrator system (Metrohm 888 Titrando, Metrohm AG, Herisau, Switzerland) was calibrated against certified reference materials (Professor A.G. Dickson, Scripps Institution of Oceanography, San Diego, CA, USA, batch number 171). Water samples were collected three times for each replicate tank: once target temperature and pH_NBS_ conditions were achieved, and after one and two weeks of acclimation. Finally, *p*CO_2_ was calculated by inputting pH_NBS_, temperature, salinity, and A_T_ into CO2SYS^54^ alongside K1 and K2 constants by Mehrback and colleagues refit by Dickson and Millero^57,58^ and KHSO_4_ by Dickson.

### Behavioural assays

Lateralisation was tested using a detour test in a two-way T-maze (69 cm long by 21 cm wide). Sharks were tested under ambient conditions because behavioural responses to high *p*CO_2_ persist during acute exposure to ambient conditions^7^. After a five-minute habituation, turning direction was scored as sharks exited the maze. Ten turns were recorded at either side of the maze – to account for potential asymmetry of the maze – totalling 20 turning decisions per shark. The relative lateralisation index (*L*_R_; turning preference scored from − 100 to 100, where positive *L*_R_ indicates a right turning bias) and absolute lateralisation index (*L*_A_; strength of lateralisation from 0–100) were calculated as *L*_R_ = [(right turns – left turns)/sum of turns]·100, and *L*_A_ =|*L*_R_|^6^.

Volitional activity levels were quantified using accelerometers (G6A + , Cefas Technology Limited, Suffolk, UK). Accelerometers were uniformly mounted on the right side of the first dorsal fin as described in Bouyoucos et al.^21^. Sharks were tagged by 0900 each day of testing and were then isolated in individual holding tanks under treatment conditions. Prior to deployment, accelerometers were rotated through each axis for calibration^43^. Tags recorded acceleration at 25 Hz, and dynamic acceleration was separated from raw acceleration data using a two-second running mean in Igor Pro (WaveMetrics Inc., Lake Oswego, OR, USA)^51^. Overall dynamic body acceleration (ODBA) was calculated as the sum of absolute values of dynamic acceleration in each axis^43^. Activity level was quantified as the mean ODBA recorded from 1100–1500, which is enough time (i.e., two hours) for juvenile sharks to resume consistent activity after capture, handling, and tagging^59^. Accelerometers weighed 5.2 g in water (i.e., a 9–18% increase in sharks’ apparent submerged weight) and had a frontal area of 476 mm^2^ (i.e., a 10–19% increase in sharks’ frontal area). Tag burden should be assessed in future studies, as it has implications for the accuracy of ODBA data^42^.

### Physiological assays

To quantify hypoxia tolerance, sharks were tested individually in a circular pool (100 L, 1 m diameter) under treatment conditions. After five minutes of habituation, oxygen saturation was lowered (8.9 ± 2.4% air saturation min^−1^) by bubbling nitrogen gas into the water. Oxygen saturation was monitored continuously with a Firesting Optical Oxygen Meter (PyroScience GmbH, Aachen, Germany). The onset of muscle spasms (OS) was used as a non-lethal experimental endpoint^21^; the oxygen saturation at OS was recorded to quantify hypoxia tolerance. Sharks were immediately returned to their treatment tank at the conclusion of the test.

Oxygen uptake rates (*Ṁ*O_2_, mg O_2_ kg^−0.89^ h^−1^) were quantified using intermittent-flow respirometry^60^. Sharks underwent a single respirometry trial to measure their minimum *Ṁ*O_2_ (*Ṁ*O_2Min_) and maximum *Ṁ*O_2_ (*Ṁ*O_2Max_). To accomplish this, sharks were first exercised (three minutes of chasing and one minute of air exposure) in a pool (100 L, 1 m diameter) under treatment conditions to achieve *Ṁ*O_2Max_ immediately post-exercise^60^. Sharks were then transferred to the same respirometry system described by Bouyoucos et al.^27^ for 24 h of *Ṁ*O_2_ determinations (n = 96) to achieve *Ṁ*O_2Min_^61^. Following respirometry, sharks were weighed and returned to their treatment tank. Background *Ṁ*O_2_ (*Ṁ*O_2Background_) was measured in empty chambers immediately before and after respirometry with sharks.

Briefly, *Ṁ*O_2_ was calculated as the absolute value of the slope of the linear decline in dissolved oxygen (mg O_2_ L^−1^ s^−1^, extracted using custom R code; A. Merciere & T. Norin, unpublished data) with a coefficient of determination greater than 0.95 during each determination and corrected by the volume of water in respirometry chambers. Because of variation in shark mass (range = 0.7–1.4 kg), *Ṁ*O_2_ was allometrically scaled to 1 kg using a mass-scaling coefficient of 0.89^23^. Shark *Ṁ*O_2_ was corrected for background respiration by fitting a line to the two *Ṁ*O_2Background_ measurements and subtracting the interpolated value from each *Ṁ*O_2_ determination^60^. Six *Ṁ*O_2_ metrics were then calculated. First, *Ṁ*O_2Min_ (1) was calculated with the Mean of the Lowest Normal Distribution method outlined by Chabot et al.^61^. Next, *Ṁ*O_2Max_ (2) was calculated from the highest slope measured over consecutive 30 s intervals during the first hour of respirometry^27^. Both absolute aerobic scope (AAS = *Ṁ*O_2Max_–*Ṁ*O_2Min_; 3) and factorial aerobic scope (FAS = *Ṁ*O_2Max_·*Ṁ*O_2Min_^−1^; 4) were calculated. Excess post-exercise oxygen consumption (EPOC, mg O_2_ kg^-0.89^; 5) was calculated as the area bound by an exponential decay curve fit to *Ṁ*O_2_, *Ṁ*O_2Min_, and the intersection of these curves^27^; this intersection was recorded as sharks’ recovery time (6) following exercise.

Blood samples (1 mL) were collected immediately after removing sharks from respirometry^27^. Samples were collected using caudal puncture with 23-gauge, heparinised needles. Whole blood pH (1) was measured with a temperature-correcting pH meter (HI98165, Hanna Instruments, Victoria, Australia) and correction equations for subtropical sharks^62^. Whole blood lactate concentration (mmol L^−1^; 2) was measured with an Accutrend Plus (Roche Diagnostics Ltd., Rotkreuz, Switzerland)^27^. Blood samples were centrifuged (10,000 g for three minutes) in duplicate to measure haematocrit (Hct, %; 3). Haemoglobin (Hb) concentration ([Hb], mmol L^−1^; 4) was measured by incubating 5 µl of whole blood in 1 mL of Drabkin’s reagent (potassium cyanide – potassium ferricyanide; Sigma-Aldrich, St. Louis, MO, USA) for at least 15 min and measuring absorbance of 200 µl aliquots in triplicate in a 96 well plate. Absorbance was read at 540 nm and [Hb] was calculated with an extinction coefficient of 11 mmo L^−1^ cm^−1^^63^. Finally, mean corpuscular haemoglobin concentration (MCHC, mmol L^−1^; 5) was calculated as [Hb]·Hct^−1^.

### Statistical analyses

Behavioural and physiological assays yielded 15 metrics: *L*_R_, *L*_A_, ODBA, hypoxia tolerance, *Ṁ*O_2Min_, *Ṁ*O_2Max_, AAS, FAS, EPOC, recovery time, whole blood pH, whole blood lactate concentration, Hct, [Hb], and MCHC. All metrics were fit with linear mixed effects models assuming Gaussian distributions using the R package ‘lme4′^64,65^, with temperature and *p*CO_2_ as interacting nominal fixed effects and replicate group as a random effect. Whilst model assumptions were met, linear mixed effects models are generally robust to violations of distributional assumptions^66^. Models including interaction terms were compared against nested models without interaction terms to estimate power to detect interactions using the ‘simr’ R package^24^. Models with interaction terms were only tested if power was > 80%. Then, the observed power of significant treatment effects could be estimated for the resulting models. Significance of fixed effects was determined by generating 95% confidence intervals (CI) of fixed effect estimate distributions from 1000 posterior simulations that were run using the R package ‘arm’^67^.

An additional series of analyses were conducted for lateralisation metrics. Frequency distributions of *L*_R_ were compared between treatments with Kolmogorov–Smirnov tests^6^. Variances of *L*_R_ and *L*_A_ were compared between treatments with Bartlett tests of homogeneity of variances^6^.

Power analysis was used to estimate the number of replicate groups needed to confidently test for effects of temperature, *p*CO_2_, and their interaction on *Ṁ*O_2Min_ using a simple 2 × 2, fully factorial design. For this analysis, we used our model for *Ṁ*O_2Min_ of blacktip reef shark neonates from the first objective. The number of replicate groups in each model was increased using the ‘simr’ R package until increases in power plateaued above 80%. Then, we specified expected temperature, *p*CO_2_, and interaction effect sizes in our models using generic estimates from Lefevre’s meta-analysis of climate change effects on *Ṁ*O_2Min_ in marine ectotherms^1^. A mean effect size for each variable was calculated using available data from all marine taxa in the meta-analysis, which included teleost (*n* = 13 species) and elasmobranch (*n* = 1) fishes. Calculated effect sizes (i.e., ratios of measured values for treatment *vs* control) were 1.67 for temperature, 1.06 for *p*CO_2_, and 1.60 for their interaction.

Coefficients of variation (CV, %) were calculated for each metric to quantify inter-individual variability. Then, associations between all traits were tested for using Pearson’s correlation tests. Statistically significant correlations were determined with a Bonferroni-corrected α = 0.0005 to account for multiple comparisons (*n* = 105).

## Data Availability

Data presented in this manuscript are available from the Research Data Repository (Tropical Data Hub) at JCU: https://dx.doi.org/10.25903/5da407f2406f5.
